# Atherosclerosis in young Brazilians suffering violent deaths: a pathological study

**DOI:** 10.1186/1756-0500-4-531

**Published:** 2011-12-12

**Authors:** Manoel ES Modelli, Áurea S Cherulli, Lenora Gandolfi, Riccardo Pratesi

**Affiliations:** 1 Department of Post Graduate School of Health Sciences, University of Brasilia, Brasília, Brazil; 2 Institute of Legal Medicine of the Federal District, Brasília, Brazil

**Keywords:** atherosclerosis, sudden death, young, pathological study

## Abstract

**Background:**

Atherosclerosis is the leading cause of coronary heart disease and ischemic stroke, which can cause sudden death in adulthood. In general, the clinical manifestations of cardiovascular diseases are caused by atherosclerosis, which is a process that starts during middle age. More recent studies indicate that the atherosclerotic process begins during childhood.

**Methods:**

To evaluate the extent of atherosclerotic disease in young Brazilians, we conducted a study of the pathological alterations in the major arteries of victims of violent death. Samples of the right carotid artery, left coronary artery, and thoracic aorta of young victims of violent death were analyzed and graded in accordance with the histological atherosclerotic lesion types proposed by the American Heart Association. Samples were collected from 100 individuals who had died from external causes, aged from 12 to 33 years.

**Results:**

The majority of cases (83%) were male, and 66% of deaths were homicides caused by firearms. The median age was 20.0 years and mean body mass index was 20.9 kg/m^2^. Of the right carotid artery specimens, 3% were normal, 55% had type I, 40% had type II, 1% had type III, and 1% had type IV atherosclerotic lesions. Of the left coronary artery specimens, 5% were normal, 48% had type I, 41% had type II, 3% had type III, and 3% had type IV lesions. Of the thoracic aorta specimens, none were normal, 13% had type I, 64% had type II, 22% had type III, and 1% had type IV lesions. Overall, 97.34% of arteries examined had some degree of atherosclerosis. The most common histological type was type II (foam cells). No thoracic aorta specimens were normal, and the coronary artery specimens had the most atherosclerosis.

**Conclusions:**

Our results show a high prevalence of atherosclerotic lesions among young people in Brazil. Intervention should be undertaken to decrease the rate of sudden cardiac death in the adult population.

## Background

Cardiovascular diseases are a major cause of death in developed and developing countries, with a significant medical, social, and economic impact [1]. In general, the clinical manifestations of cardiovascular diseases, such as myocardial infarction, stroke, and peripheral vascular disease, are caused by an atherosclerotic process that is initiated during middle age. Sudden death of cardiac origin, defined as death which occurs within 6 hours of onset of symptoms or within 24 hours of the victim having been seen in a normal state of health, is responsible for approximately 50% of all deaths of cardiovascular origin [2].

Sudden death may be the first and only manifestation of arterial disease. Sudden death due to coronary artery disease is the cause of death in 60-70% of autopsies, occurring most frequently among men aged 50-70 years [3]. In children, the most common pathological causes of sudden death are myocarditis, hypertrophic cardiomyopathy, congenital coronary anomaly, coronary artery disease, conduction system abnormalities, and mitral valve prolapse [4].

Several long-term epidemiological studies of cardiovascular risk factors have been undertaken, such as The Bogalusa Heart Study (BHS) in Louisiana which was initiated in 1973 [5], The Muscatine in Ohio [6], and the Pathological Determinants of Atherosclerosis in Youth (PDAY) Research Group in Louisiana [7]. In the BHS, all study subjects had fatty streaks in the aorta and coronary arteries, the prevalence of which increased with age. Autopsy studies of youths have established a strong association between cardiovascular risk factors and early stages of coronary atherosclerosis such as intimal thickening, as assessed using ultrasound [8].

The etiology of atherosclerosis causing coronary heart disease is complex. Pathogenesis includes hemodynamic and thrombotic factors, high levels of cholesterol, smoking, lifestyle, and intrinsic factors of the arterial wall. An excessive inflammatory response to various insults to the endothelium and smooth muscle of the arterial wall results in lymphoproliferation and injury. A large number of growth factors, cytokines, and molecules are involved in this process [9,10].

Current studies indicate that atherosclerosis begins to develop in childhood, with fatty streaks grossly visible in the aortas of children from 3 years of age [11]. These findings have changed the model of atherosclerosis as a chronic degenerative disease of elderly patients to a model of subclinical chronic inflammatory disease starting in childhood and influenced by known risk factors, autoimmune reactions, and more recently by infectious agents such as *Chlamydia pneumoniae *which are currently the focus of numerous studies [12].

The aim of our study was to obtain a profile of atherosclerotic changes among young people of the Federal District (Brasilia-Brazil) by histopathological analysis of three major arteries in victims of violent death, and to evaluate the relationship between atherosclerotic changes and age, sex, body mass index, and heart weight.

## Methods

### Subjects

This prospective observational study included 100 victims of violent death who were autopsied at the Institute of Forensic Medicine of the Federal District, Brasilia from August 2008 to December 2009. The group studied was of apparently healthy young people, chosen at random. The study was approved by the Research Ethics Committee of the Faculty of Health Sciences, Federal District (DF-FEPECS, project number 125/2008). The Research Ethics Committee waived the requirement for informed consent because a complete cadaver examination (including macroscopic and microscopic analysis) is mandatory in all cases of violent death according to the laws of the country.

### Samples collected

Samples were taken from the right common carotid artery, left coronary artery, and proximal 10 cm of the thoracic aorta. All samples were 1 cm in length, and were fixed in 10% formalin and stained with hematoxylin and eosin. The samples were all analyzed by the same pathologist, and histopathological changes were graded according to the classification proposed by the Committee on Vascular Lesions of the Council on Arteriosclerosis, American Heart Association into six lesion types: type I (intimal thickening), type II (presence of foam cells), type III (small deposits of extracellular lipid-fatty streaks), type IV (atheroma), type V (fibroatheroma), and type VI (defect on the surface of the intima, with the occurrence of bleeding and clotting) [13].

### Data collected

The following data were analyzed for each case: sex, age, height, body mass, body mass index, and heart weight.

Statistical analysis: Statistical analysis was undertaken to investigate the association between atherosclerosis and sex, age (Mantel-Haenszel chi-square test), heart weight, and body mass index (Kruskal-Wallis test). Statistical significance was set at p < 0.05 [14].

## Results

**Figure 1:**
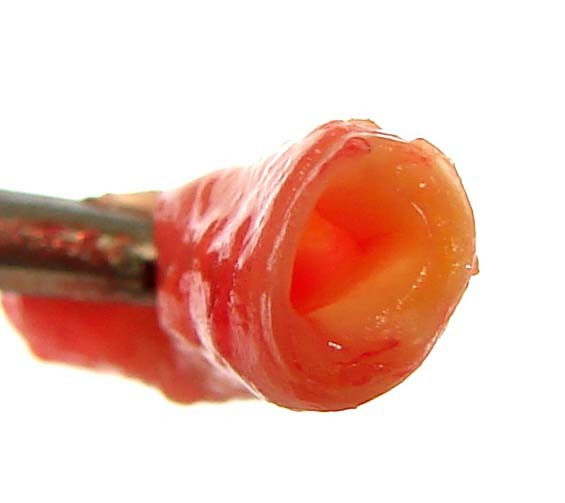
**Atherosclerotic plaque in a coronary artery**.

**Figure 2:**
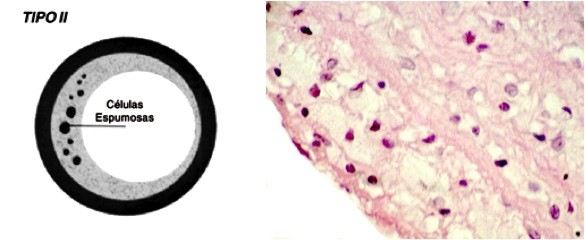
**Type II atherosclerotic lesion (hematoxylin and eosin, 400×)**.

Of a total of 300 samples of arteries, 97.34% had an identifiable atherosclerotic lesion (Figure 1), with type II lesions (foam cells) being the most common (Figure 2). No sample of the aorta was normal. Among the 100 cases examined, the age ranged from 12 to 33 years with a mean age ± standard deviation (SD) of 20.12 ± 4.08 years (mode 16 years), and the majority were male (83%). All cases were victims of violent death, with 66% caused by firearms. The average interval between death and autopsy was 12.8 ± 4.4 h, with all cases autopsied within 24 h after death. The mean body mass index was 20.95 ± 3.2 kg/m^2 ^(mode 17.5 kg/m^2^). The mean heart weight was 280 ± 56.3 g (mode 300 g). The distribution of histological lesions in the arteries is shown in Table 1.

**Table 1:** Histopathological changes in the arteries of young victims of violent death.

Arteries	Normal	Grau I	Grau II	Grau III	Grau VI
**Carotid**	3	55	40	1	1
**Coronary**	5	48	41	3	3
**Aorta**	0	13	64	22	1

Results of the Mantel-Haenszel chi-square test suggest that males tended to have more advanced lesion types in the carotid arteries than females, but this was not statistically significant (p = 0.0781). Heart weight tended to correlate with severity of atherosclerotic lesions in the carotid arteries, but this association was also not significant. Results of the Kruskal-Wallis test showed that body mass index tended to be associated with the severity of atherosclerosis in the aorta, but this association was not significant. The only significant association in our cohort was between age and the severity of lesions in the carotid arteries, with the chi-square Mantel-Haenszel test showing increased severity in patients older than 20 years (p = 0.0388).

## Discussion

Of all 300 arterial samples, 97.34% showed some degree of atherosclerotic lesion. Our results are consistent with previously published data indicating atherosclerotic involvement of the main arteries in young populations, but the prevalence in our study is higher [15]. The data show a significant impairment of the arteries investigated, especially of the aorta, where there were no cases with normal histology. However, the most advanced lesions were found in the coronary arteries.

Atherosclerotic lesions in children were first discussed by Hodgson in 1815 [16]. Two studies of autopsies on US soldiers focused on atherosclerotic lesions in the arteries of young subjects. The first was conducted by Enos *et al *(1953) during the Korean War and found that 77.3% of coronary arteries studies had changes [17], and the second was conducted by McNamara *et al *(1971) during the Vietnam War and found that 45% of subjects had some evidence of coronary atherosclerosis [18].

Type I and II lesions, being the earliest lesions, usually appear within the first three decades of life, and type III lesions predominate during the following two decades. During the first 5 years of life, usually only type I lesions are found. Stary *et al. *[19] noted no significant differences in prevalence with sex or skin color in the younger age groups, although blacks develop lesions at a younger age.

Studies examining the histopathological aspects of early and advanced atherosclerotic lesions have emphasized the crucial role of macrophages in the formation of foam cells and fatty streaks. Atherosclerotic lesions are considered advanced when there is accumulation of cells, lipids, and matrix components including minerals, and when they are associated with structural disorganization, repair and thickening of the intima, and deformity of the arterial wall [20,21].

In 1990, the PDAY study group published data assessing the aorta and right coronary artery in 390 males aged 15 to 34 years, which showed that fatty streaks begin earlier in the thoracic and abdominal aorta than the right coronary artery, and are therefore more prevalent in the aorta, but that coronary artery lesions tend to be more advanced, especially in older age groups. They also suggested that the tendency of fatty streaks to progress to more advanced lesions varies according to the injury site [22].

Similar results were obtained by Strong *et al *[23] in a study of autopsies of young people, which found intimal lesions appeared in all the aortas and in more than half the coronary arteries in the group aged 15 to 19 years, with a higher prevalence in the older group.

We found that thoracic aortas were compromised in all patients, and that the most advanced lesions were present in coronary arteries. In autopsy studies, Dalager *et al *[24] found distinct patterns of involvement in carotid, coronary, and femoral arteries, and concluded that femoral artery plaques are formed later than coronary and carotid artery plaques. Unlike carotid artery plaque, femoral artery plaque was significantly associated with cardiac death.

The involvement of other arteries in the body also has been investigated by Seo *et al *[25] who found that 95.6% of Korean women had pathological changes, and by Restrepo *et al *[26] who compared male victims of violent death from New Orleans and Guatemala. Involvement of the cerebral arteries is a common cause of ischemic stroke, mainly by obstruction of the middle cerebral artery. In the circle of Willis, the prevalence of cerebral artery involvement is 76.5% in the fourth decade and 87.5% in the fifth decade, increasing to 100% at older ages [27].

Mc Mahan *et al *[28] investigated the relationship of coronary risk factors (sex, age, lipid levels, smoking, hypertension, obesity, and hyperglycemia) with early atherosclerotic lesions in individuals between 15 and 34 years old, who died of external causes and underwent autopsy by forensic medicine services. They found that risk factors were associated with type I lesions of the left main coronary artery and fatty streak lesions of the right coronary artery and abdominal aorta. These data support changing the lifestyles of young people to prevent the development of early lesions and thereby prevent heart disease later.

Besides high levels of serum lipids, infections and genetic susceptibility have been implicated as possible etiologic factors for the development of atherosclerotic lesions of coronary arteries in children. Recently, there has been some discussion whether feeding infants formula rather than breast milk and parental smoking may be major causes of atherosclerosis in children.

According to United Nations Children's Fund (UNICEF), infant formulas are responsible for 1.5 million child deaths each year in the US. The consequences of this type of feed include metabolic disorders, delayed development of the nervous system, and dyslipidemia. Studies have shown an association between maternal smoking and sclerotic changes in the coronary arteries of fetuses after 35 weeks of gestation [29].

## Conclusions

Even though Brazil is a developing country, the prevalence of atherosclerosis is extremely high, with 97.34% of arterial samples of young people showing changes. In recent decades, Brazilian people have acquired habits of the developed world, including fast-food eating habits, which seems to be reflected in their current rates of atherosclerotic disease and consequently sudden death, the majority of which is of cardiac origin. Brazil is therefore a developing country with the sudden cardiac death rate of developed countries.

The most important result of our study was the demonstration of atherosclerosis in the major arteries of young individuals. The subjects chosen for this study were apparently healthy people who were victims of violent deaths, thus avoiding people with chronic diseases.

## Study limitations

Statistical analysis was compromised by the small sample size, and some conclusions did not attain a satisfactory level of significance, although they represent an important warning of the prevalence of atherosclerosis early in life.

This is only a pathological study showing that atherosclerosis is common in young Brazilians, and drawing attention to the importance of early prevention.

## Competing interests

The authors declare that they have no competing interests.

## Authors' contributions

MM participated in the study design, collected the samples, and drafted the manuscript. AC was the pathologist responsible for the histopathological analysis of the samples. LG participated in the study design and helped to draft the manuscript. RP participated in the study design and helped to draft the manuscript. All authors read and approved the final manuscript.
